# Reperfusion Into Severely Damaged Brain Tissue Is Associated With Occurrence of Parenchymal Hemorrhage for Acute Ischemic Stroke

**DOI:** 10.3389/fneur.2020.00586

**Published:** 2020-06-26

**Authors:** Songlin Yu, Samantha J. Ma, David S. Liebeskind, Xin J. Qiao, Lirong Yan, Jeffrey L. Saver, Noriko Salamon, Danny J. J. Wang

**Affiliations:** ^1^Department of Neurosurgery, Beijing Tiantan Hospital, Capital Medical University, Beijing, China; ^2^Neurovascular Imaging Research Core and UCLA Stroke Center, Department of Neurology, UCLA, Los Angeles, CA, United States; ^3^CAS Center for Excellence in Brain Science and Intelligence Technology, Chinese Academy of Sciences, Shanghai, China; ^4^Department of Neurology, USC Stevens Neuroimaging and Informatics Institute, USC, Los Angeles, CA, United States; ^5^Department of Radiology, UCLA, Los Angeles, CA, United States

**Keywords:** acute ischemic stroke (AIS), arterial spin labeling (ASL), reperfusion, reperfusion injury, hemorrhagic transformation (HT)

## Abstract

**Background and Purpose:** This study aims to quantify the reperfusion status within severely damaged brain tissue and to evaluate its relationship with high grade of hemorrhagic transformation (HT).

**Methods:** Pseudo-continuous ASL was performed along with DWI in 102 patients within 24 h post-treatments. The infarction core was identified using ADC values <550 × 10^−6^ mm^2^/s. CBF within the infarction core and its contralateral counterpart were acquired. CBF at the 25th, median, and 75th percentiles of the contralateral counterpart were used as thresholds and the ASL reperfusion volume above the threshold was labeled as vol-25, -50, and -75, respectively. Recanalization was defined according to Thrombolysis in Myocardial Infarction (TIMI) criteria.

**Results:** Quantified reperfusion within the infarction core differed significantly in patients with complete and incomplete recanalization. In the ROC analysis for the prediction of parenchymal hematoma (PH), ASL reperfusion vol-25 had the highest area under the curve (AUC) when compared with ASL vol-50 and ASL vol-75. ASL reperfusion vol-25 had significantly higher AUC compared with ADC threshold volume in the prediction of PH (0.783 vs. 0.685, *P* = 0.0036) and PH-2 (0.844 vs. 0.754, *P* = 0.0035). In stepwise multivariate logistic regression analysis, only ASL reperfusion vol-25 emerged as an independent predictor of PH (OR = 3.51, 95% CI: 1.65–7.45, *P* < 0.001) and PH-2 (OR = 2.32, 95% CI: 1.13–4.76, *P* = 0.022).

**Conclusions:** Increased reperfusion volume within severely damaged brain tissue is associated with the occurrence of higher grade of HT.

## Introduction

Acute ischemic stroke (AIS) is a leading cause of death and disability worldwide. Currently, intravenous infusion of tissue plasminogen activator (tPA) is the main therapy with proven clinical benefit for ischemic stroke (1, 2). More recently, endovascular therapy has been shown to benefit a subgroup of AIS patients with specific neuroimaging and/or clinical profiles (3–10). As a result, increased options for thrombolytic and revascularization therapy are available with improved rate of reperfusion and clinical outcome of AIS patients. However, the details regarding post-treatment care remain unspecified. Reperfusion in time can reduce more extensive brain tissue injury by salvaging a reversibly damaged penumbra of tissue. On the other hand, paradoxically, it also carries the risk of causing additional and substantial brain damages, such as ischemia-reperfusion injury, if recanalization occurs too late, compared with no revascularization (11).

Ischemia-reperfusion injury has been demonstrated in a variety of organ systems in animal models due to many potential mechanisms, such as inflammation and excessive reactive oxygen species (ROS) generation, which can disrupt the neurovascular unit and increase blood brain barrier (BBB) permeability, thus leading to hemorrhagic transformation (HT) (12, 13).

Parenchymal hematoma (PH) is the most feared and potentially life-threatening complication of revascularization therapy in patients with AIS (14). Other studies, such as the ECASS-I and ECASS-II trials, have shown only PH type 2 (PH-2) impacted long-term outcome, which was defined as death or severe disability at 3 months after stroke (15–17). A valid method to assess the risk of hemorrhagic transformation, especially PH, would improve the safety of thrombolytic therapy.

DWI is typically considered as the reference standard for identifying infarct core. Lesions on DWI represent brain tissue that is severely damaged and more vulnerable to reperfusion injuries. After recanalization treatment, a substantial number of patients may experience reocclusion or new discrete infarctions (18). The reperfusion status after thrombolysis or endovascular treatment within the DWI lesion territory and its relationship with HT is not clear. In this research, we aim to quantify the volume of reperfusion into severely damaged brain tissue and investigate its relationship with the occurrence of parenchymal hematoma following recanalization.

## Materials and Methods

### Patient Selection

The present study was performed on data collected from June 2010 to September 2013 in a prospective registry of patients evaluated with diffusion–perfusion MRI at our academic medical center. Patients with AIS were included in this study if:(1) they were adults (aged > 18 years); (2) they had unilateral acute ischemic stroke; (3) intravenous tPA and/or endovascular therapy was performed; (4) ASL and DWI imaging were acquired within 24 h post-treatment, and (5) there was an absence of previous intracranial hemorrhage, brain surgery, or large territorial lesion. Patients with early occurrence of PH concurrent with ASL imaging were excluded. The local Institutional Review Boards approved the study.

### MRI Protocols and Analysis

All patients underwent MRI on Siemens 1.5 T Avanto or 3.0 T TIM Trio systems (Erlangen, Germany), using 12 channel head coils. ASL was performed along with other MR sequences within 24 h after treatment as a routine clinical protocol. A pseudo-continuous ASL (pCASL) pulse sequence with background suppressed 3D GRASE (gradient and spin echo) readout was applied with the following parameters: TR/TE/label time/post-labeling delay (PLD), 4,000/22/1,500/2,000 ms; field of view, 22 cm; matrix size, 64 × 64, 26 × 5 mm slices; GRAPPA factor of 2, 4/8 partial k-space, 30 pairs of label and control images with a scan time of 4 min. In December 2011, a pCASL with GRASE readout protocol with four PLDs (1,500, 2,000, 2,500, 3,000 ms) was implemented and the present analysis included CBF data obtained with the PLD of 2,000 ms. The imaging parameters of the 4-PLD ASL protocol were the same as those of the single PLD protocol except that eight pairs of label and control images were acquired for each PLD, resulting in a scan time of 4 min 30 s, as described previously (19, 20).

Data analysis was performed with Interactive Data Language [IDL (Boulder, CO, USA)] software programs developed in-house. Motion correction was performed on ASL images. Pairwise subtraction between label and control images was performed followed by averaging to generate the mean difference image. Quantitative CBF maps were calculated based on a previously published model (19). CBF maps were co-registered with DWI and normalized into the Montreal Neurological Institute template space using SPM8 (Wellcome Department of Cognitive Neurology, UCL, UK).

A 1–3 Likert-type scale was used by two reviewers respectively to evaluate the ASL image quality with scoring as follows: (1) severe image artifact (e.g., head motion), not interpretable; (2) fair diagnostic image quality, some distortion and noise, acceptable delineation of major structures; and (3) good image quality, no to minimal distortion with detailed delineation of all structures. ASL images with an image quality score of 1 by both reviewers were discarded.

Apparent diffusion coefficient (ADC) values between 200 and 1,200 × 10^−6^ mm^2^/s were selected to distinguish from non-cerebral tissues. The infarction core was identified using ADC values <550 × 10^−6^ mm^2^/s (21). If parenchymal hemotoma (PH, high grade) occurred concurrently with ASL, patients were excluded from this study. However, if hemorrhagic infarction (HI, low grade) existed, we applied a bleed ROI to exclude these tiny bleeding areas for subsequent analysis. CBF within the infarction core and its contralateral counterpart was acquired. CBF at the 25th, median, and 75th percentiles of the contralateral part was documented and used as the threshold to define reperfusion status in the lesion side, respectively. Hypoperfusion was defined as CBF values less than or equal to the threshold value at the 25th percentile, and hyperperfusion was defined as CBF values greater than the threshold value at the 75th percentile according to our previous study (22). ASL reperfusion volume above the threshold was calculated and noted as vol-25,−50, and−75, respectively. Relative reperfusion volume was defined as ASL reperfusion vol-25 divided by ADC thresholded volume.

Recanalization was defined according to thrombolysis in myocardial infarction (TIMI) criteria, dichotomized into presence (TIMI 2–3) or absence (TIMI 0–1) of recanalization, and evaluated on time-of-flight MR angiography or digital subtraction angiography (DSA) by the two raters.

Qualitative hyperperfusion was defined as patchy areas with visually perceivable increased CBF on ASL maps within the corresponding lesion observed on DWI images when compared with the homologous contralateral hemisphere (23). Follow up gradient recalled echo (GRE) scans were used to grade HT which was categorized as HI (low grade) or PH (high grade) according to published criteria (16). PH was further divided into PH1 with hematoma in ≤ 30% of the infarcted area with some slight space-occupying effect and PH2 with hematoma in >30% of the infarcted area with a substantial space-occupying effect. The two raters were blinded to clinical information and independently reviewed the MRIs to determine the types of HT on GRE.

Any discrepancies with regard to the presence of HT grades and recanalization status between the raters were resolved by consensus agreement. In-hospital death or survival and functional outcome at 3 months were recorded. Good functional outcome was defined as a 90-day modified Rankin Scale (mRS) score of 0–2.

### Statistical Analysis

Statistical analysis was performed using SPSS (IBM SPSS Statistics, Armonk, NY) and R (R Core Team (2012) Vienna, Austria). The weighted *kappa* test was used to evaluate the inter-rater agreement in terms of ASL imaging quality. Paired-Samples *T*-test was used to investigate if there was a significant difference between CBF value in the DWI lesion territory and its non-lesion counterpart. The median ASL reperfusion volume and ADC threshold lesion volume in each HT category were compared using the non-parametric Mann–Whitney *U*-test. Tests were conducted using a Bonferroni adjusted alpha of 0.0125 per test (0.05/4). Receiver operating characteristic (ROC) analysis was performed. The area under the ROC curve (AUC) was used as a scalar measure to assess the performance of prognostic risk scores. The AUC between groups was compared by using the Delong et al. (24) method. The data was then transformed (by the fifth root) to satisfy the assumption of normal distribution. Univariate logistic regression analysis was performed to evaluate the predictive value of the imaging and clinical variables for binary PH vs. no PH and PH2 vs. no PH2. The Bonferroni adjusted alpha of 0.003125 per test (0.05/16) was used to determine significance. Subsequently, multiple logistic regression (stepwise) analysis was applied to identify predictors for HT categories from the variables that had a univariate probability value <0.2. The stepwise analyses (forward, backward, and bi-directional) chose the best multivariate model fit by selecting variables that minimized the Akaike Information Criterion (AIC).

## Results

A total of 107 patients were included from June 2010 to 2013. Five patients were then excluded due to poor ASL imaging quality in three patients and early occurrence of PH-1 concurrent with ASL in two patients. Thus, the remaining 102 patients [age = 71.3 ± 14.5 (mean ± SD) years; 49 males] were used for analysis. The median NIHSS score was 16 (interquartile range, 9–21). The median time from treatment to ASL/DWI imaging was 6.7 (interquartile range, 4.8–18.3) h. The average score of image quality of included ASL maps was 2.63 ± 0.48. The κ coefficient was 0.75 for ASL imaging quality between the two raters.

Overall, HT affected 56/102 (55%) patients, including 27/102 (26%) HI, 16/102 (16%) PH1, and 13/102 (13%) PH2 (Table 1).

**Table 1:** Characteristics of patients by hemorrhagic transformation grade.

	**No HT**	**HI**	**PH-1**	**PH-2**
Number	46	27	16	13
Female Gender, *n* (%)	27 (59)	10 (37)	8 (50)	8 (62)
Atrial Fibrillation, *n* (%)	9 (20)	11 (41)	6 (38)	4 (31)
Hypertension, *n* (%)	30 (65)	20 (74)	10 (63)	8 (62)
Diabetes, *n* (%)	8 (17)	6 (22)	3 (19)	2 (15)
Hyperlipidemia, *n* (%)	16 (35)	12 (44)	1 (6)	6 (46)
Aspirin use, *n* (%)	12 (26)	3 (11)	4 (25)	4 (31)
Age (mean ± SD), year	71 ± 13	70 ± 18	73 ± 13	74 ± 16
NIHSS (mean ± SD)	13 ± 7	17 ± 8	15 ± 8	18 ± 4
Glucose (mean ± SD), mg/dL	133 ± 40	136 ± 57	147 ± 43	153 ± 61
Time to treatment (mean ± SD), h	4 ± 5	4 ± 4	12 ± 23	3 ± 3
**TREATMENT MODALITY**				
IV thrombolysis, *n* (%)	36 (78)	24 (89)	15 (94)	10 (77)
IA thrombolysis, *n* (%)	7 (15)	2 (7)	0 (0)	2 (15)
Clot retrieval, *n* (%)	13 (28)	9 (33)	5 (31)	8 (62)
Post-treatment TIMI 2 or 3	30 (65)	21 (78)	14 (88)	13 (100)
Visually hyperperfusion	12 (26)	11 (41)	8 (50)	7 (54)
**VOLUME ON POST-TREATMENT MR (MEAN** **±** **SD), ml**				
ADC threshold volume	38 ± 80	50 ± 98	37 ± 52	66 ± 58
ASL reperfusion vol-25	6 ± 7	10 ± 13	21 ± 36	33 ± 32
ASL reperfusion vol-50	3 ± 4	7 ± 9	15 ± 30	18 ± 17
ASL reperfusion vol-75	2 ± 4	4 ± 7	11 ± 24	10 ± 12
Relative reperfusion volume (%)	58 ± 34	53 ± 34	63 ± 26	54 ± 22
In hospital death, *n* (%)	5 (11)	2 (7)	1 (6)	5 (38)

*HT indicates hemorrhagic Transformation; IV, intravenous; IA, intra-arterial; tPA, tissue-type plasminogen activator; NIHSS, National Institutes of Health Stroke Scale; DWI, diffusion-weighted imaging; BP, blood pressure*.

Qualitative hyperperfusion was detected in 38 patients. There was a trend that patients with hyperperfusion were more likely to have PH (*P* = 0.07), although there was no significant association between having hyperperfusion and PH-2 (*P* > 0.05).

**Figure 1:**
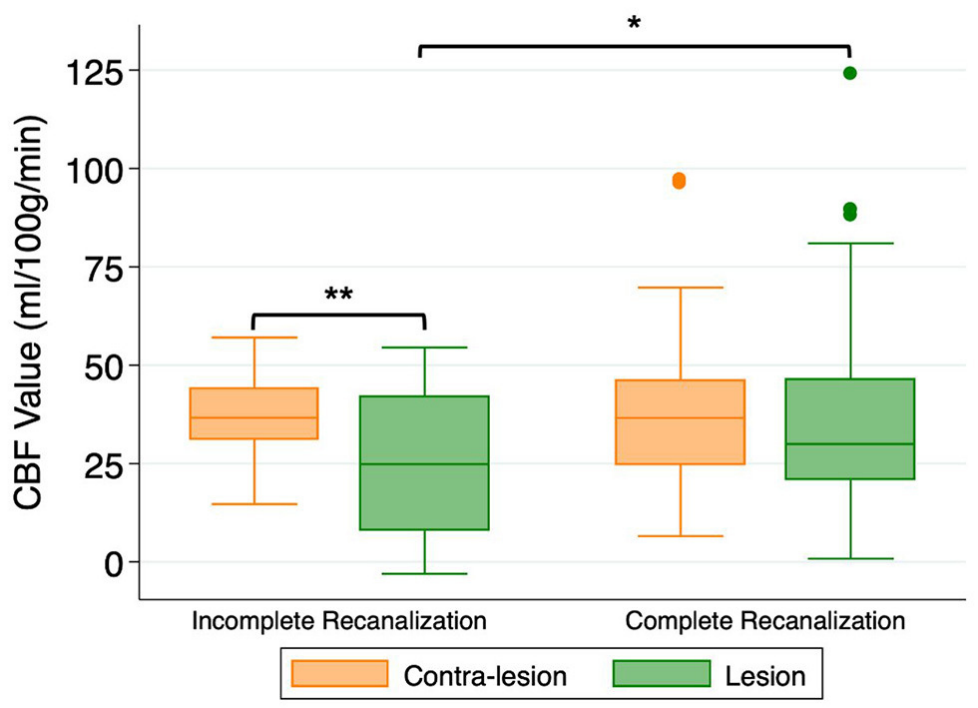
Cerebral blood flow (CBF) values within the DWI-delineated infarct core for patients with incomplete recanalization (TIMI 0–1) or complete recanalization (TIMI 2–3). ^*^P < 0.05, ^**^P < 0.01.

78/102 (76%) patients had post-treatment TIMI scores of 2–3. As shown in Figure 1, CBF within the DWI lesion territory was higher in patients with TIMI 2–3 than those with TIMI 0–1 (37.6 ± 22.8 vs. 28.2 ± 15.3 ml/100 g/min, *P* = 0.025). In patients with TIMI 0–1, CBF within the DWI lesion territory was significantly lower than in its non-lesion counterpart (25.8 ± 18.0 vs. 37.2 ± 10.7 ml/100 g/min, *P* = 0.006). By contrast, in patients with TIMI 2–3, there was no significant difference between CBF within the DWI lesion territory and in its non-lesion counterpart (37.6 ± 22.8 vs. 38.3 ± 17.2 ml/100 g/min, *P* > 0.05).

**Figure 2:**
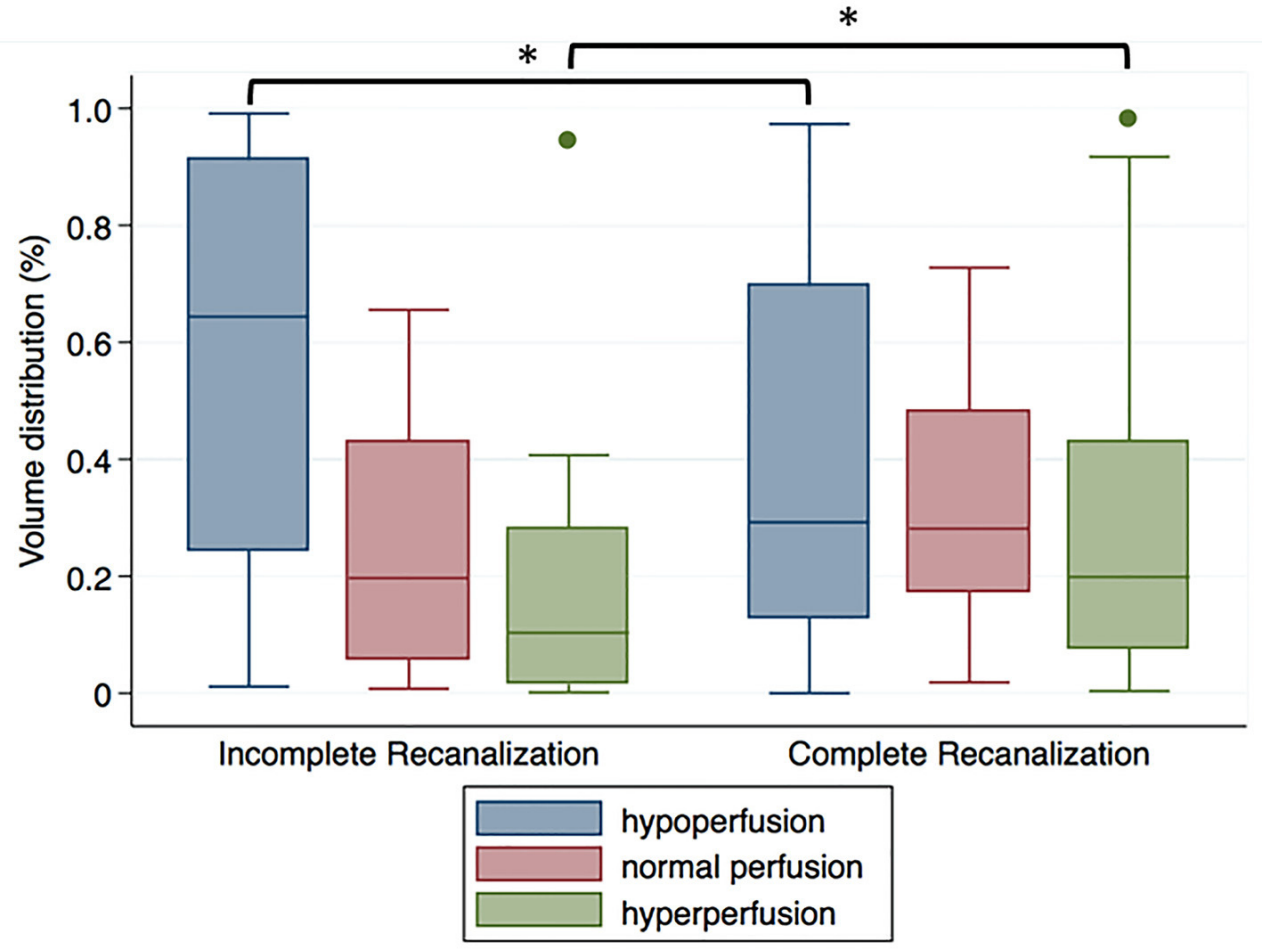
Volume distribution of different reperfusion status within the DWI-delineated infarct core for patients with incomplete recanalization (TIMI 0–1) or complete recanalization (TIMI 2–3). ^*^P < 0.05.

The median (interquartile range) CBF value used as threshold at 25th, 50th, and 75th percentile in the contra-lesional area were 26.0 (18.0–38.1), 36.0 (26.2–48.6), and 45.4 (34.1–59.2) ml/100 g/min, respectively. For severely damaged brain tissue, the percentage of volume distribution of different reperfusion status differed significantly in patients with complete and incomplete recanalization (hypoperfusion *P* = 0.02, hyperperfusion *P* = 0.02; Figure 2).

**Figure 3:**
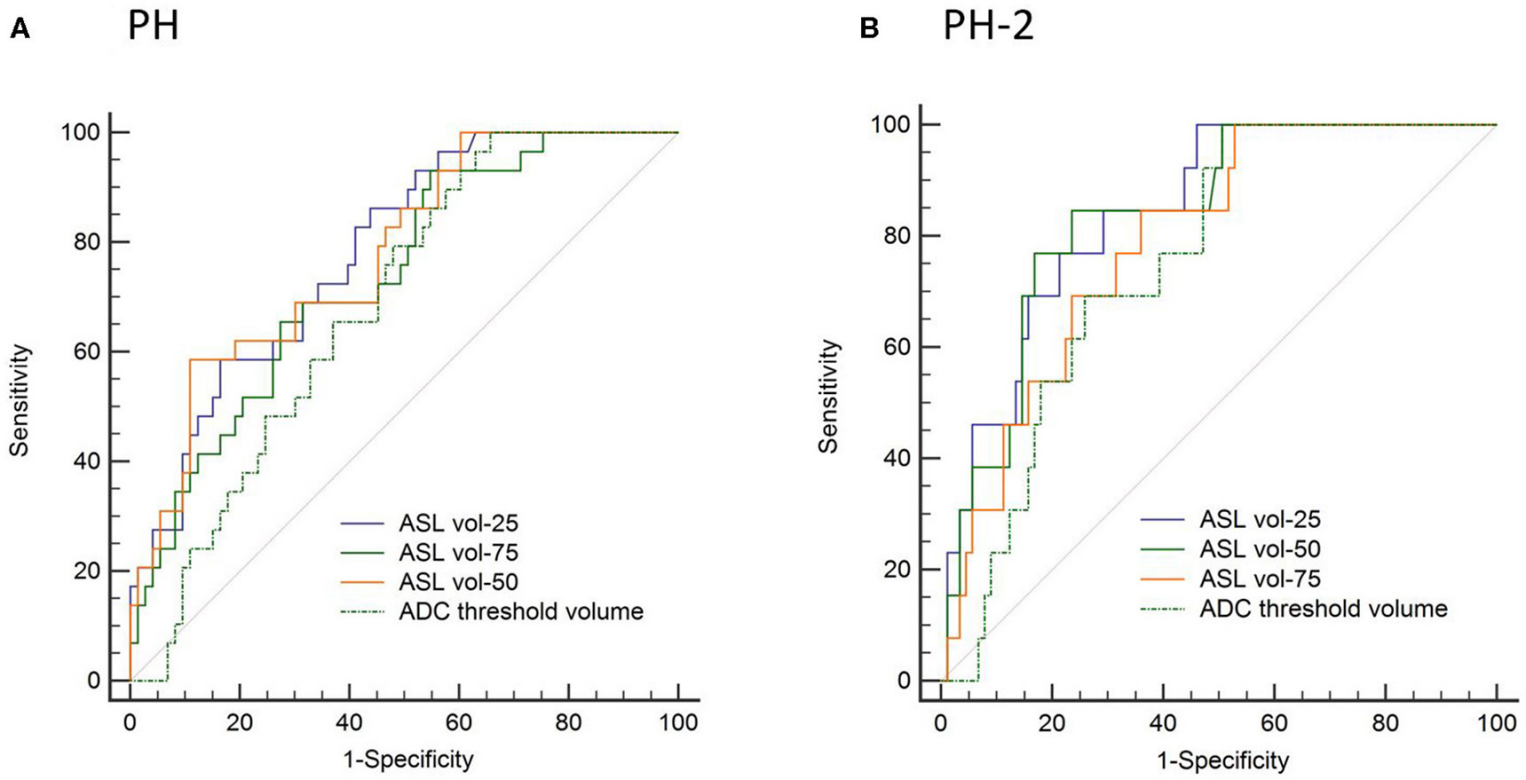
Area under the receiver operator curve (AUC) analysis in prediction of **(A)** parenchymal hematoma (PH) and **(B)** parenchymal hematoma type 2 (PH-2).

**Figure 4:**
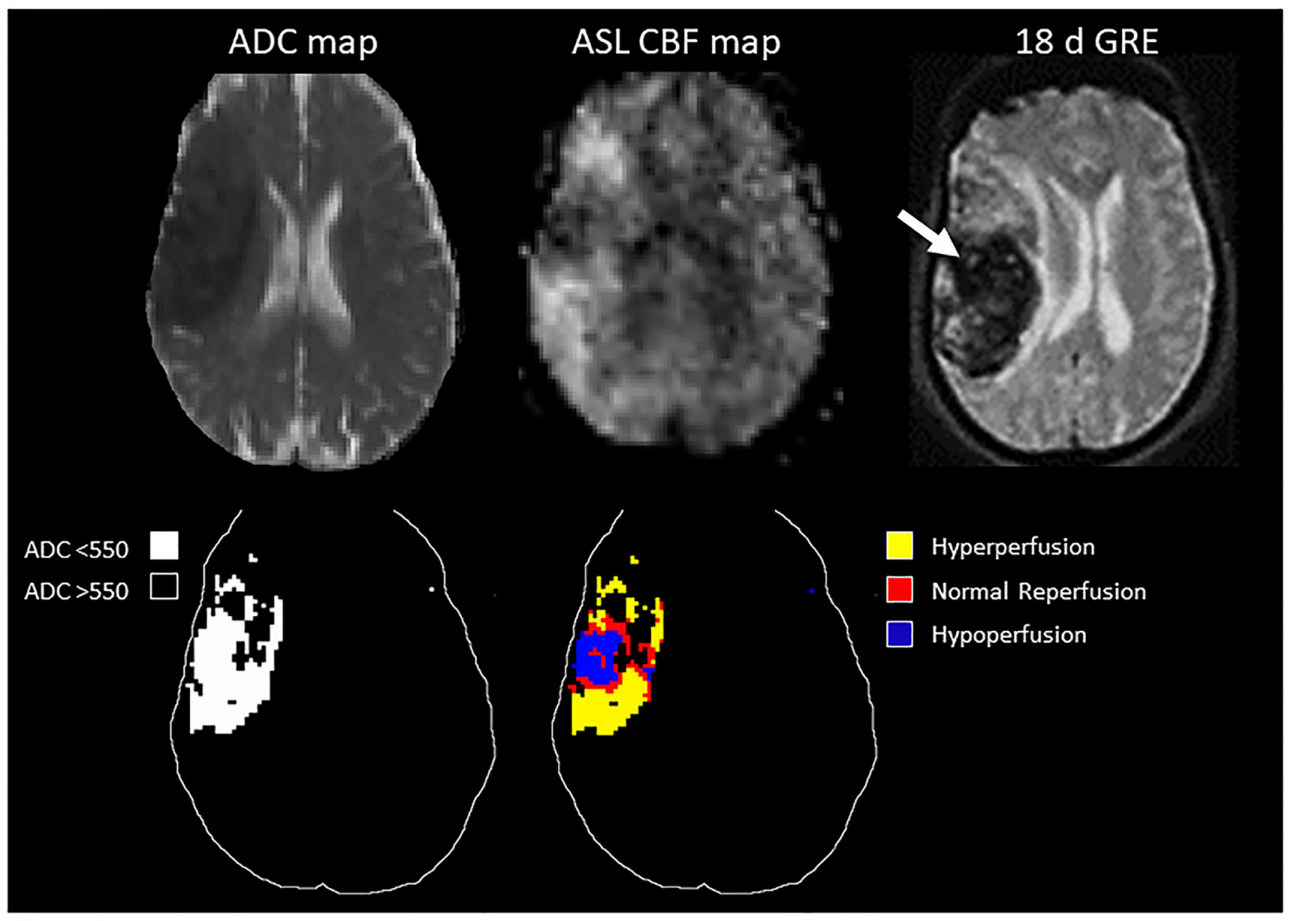
**Top:** MRI of a 78 year old patient with MCA stroke. IV tPA was given 2 h after onset, followed by clot retrieval at 4.7 h after onset. Post-treatment ASL showed variable reperfusion, and subsequent hemorrhage was observed in GRE at a later time point. **Bottom:** schematic drawing of the ADC-thresholded lesion volume, and the reperfusion volume distribution within the lesion.

**Figure 5:**
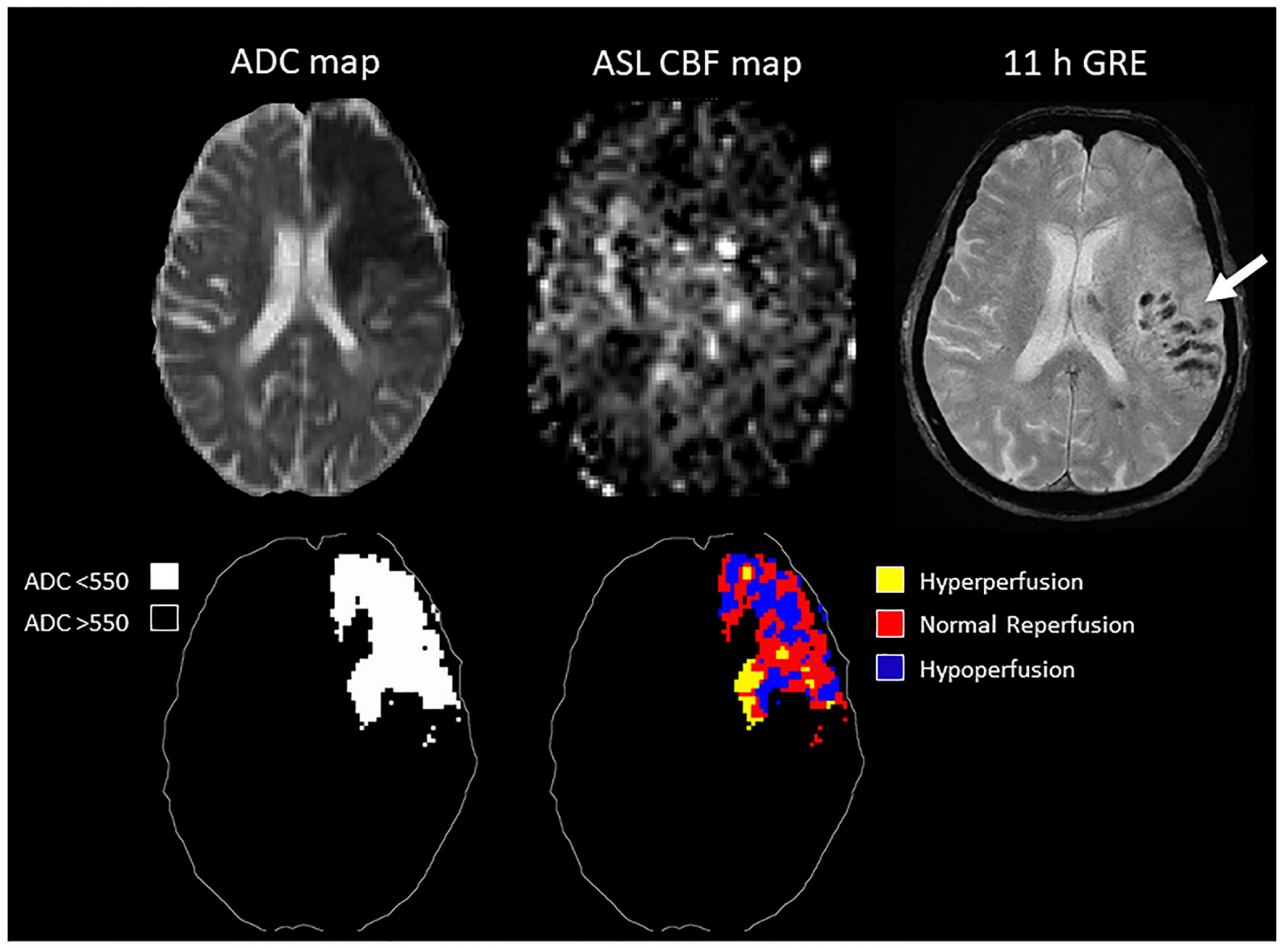
**Top:** MRI of an 89 year old patient with MCA occlusion. IV tPA was given 2 h from onset, followed by clot retrieval at 5.5 h from onset. Post-treatment ASL showed inhomogeneous reperfusion, and subsequent hemorrhage was observed in GRE at a later time point. **Bottom:** schematic drawing of the ADC-thresholded lesion volume, and the reperfusion volume distribution within the lesion.

In the ROC analysis, ASL reperfusion vol-25 had the highest area under the curve (AUC; Supplemental Table I). ASL reperfusion vol-25 had significantly higher AUC when compared with ADC threshold volume in the prediction of PH (0.783 vs.0.685, *P* = 0.0036) and PH-2 (0.844 vs. 0.754, *P* = 0.0035). ASL reperfusion vol-25 was therefore used for subsequent analysis (Figure 3). The best cut-off value for ASL reperfusion vol-25 in the prediction of PH was 4.2 ml. The best cut-off value in the prediction of PH-2 is 11.9 ml. Figures 4, 5 demonstrated the volume distributions of different reperfusion status and the subsequent hemorrhages observed in GRE at a later time point in two patients.

For the prediction of binary PH vs. no PH, univariate analysis showed that only ASL reperfusion vol-25 (*P* < 0.001) was a significant predictor considering the Bonferroni adjusted alpha (Table 2). ASL reperfusion vol-25, ADC-thresholded volume (*P* = 0.025), hyperlipidemia (*P* = 0.177), glucose (*P* = 0.145), time to treatment from symptom onset (*P* = 0.198), post-treatment TIMI score (*P* = 0.121), and clot retrieval procedure (*P* = 0.161) were considered in the multiple logistic regression analysis. In stepwise multivariate logistic regression analysis, only ASL reperfusion vol-25 (OR = 3.51, 95% CI: 1.65–7.45, *P* < 0.001) emerged as a significant predictor of PH with the non-significant albeit meaningful contributions of ADC-thresholded volume and glucose in the model. Similarly, for the prediction of binary PH2 vs. no PH2 outcome, univariate analysis showed that ASL reperfusion vol-25 (*P* = 0.001) was the only significant factor. ASL reperfusion vol-25 was considered together with ADC-thresholded volume (*P* = 0.014), clot retrieval procedure (*P* = 0.034), and baseline NIHSS (*P* = 0.086) in the multiple logistic regression analysis. Following stepwise multivariate logistic regression analysis, only ASL reperfusion vol-25 remained as an independent predictor (OR = 2.32, 95% CI: 1.13–4.76, *P* = 0.022) with non-significant contribution from clot-retrieval procedure in the model.

**Table 2:** Logistic-regression analysis by HT category and functional outcome.

**Variables**	**Univariate**			**Multivariate**		
	**OR**	**95% CI**	***P*-value**	**OR**	**95% CI**	***P*-value**
**PH vs. NO PH**						
Atrial fibrillation	1.39	0.55–3.51	0.480	–		
Hypertension	0.75	0.31–1.85	0.535	–		
Diabetes	0.88	0.28–2.71	0.821	–		
Hyperlipidemia	0.51	0.19–1.35	0.177^*^	0.48	0.14–1.63	0.241
Aspirin use	1.47	0.55–3.97	0.444	–		
Age, year	1.01	0.98–1.04	0.418	–		
NIHSS	1.04	0.98–1.10	0.222	–		
Glucose, mg/dL	1.01	1.00–1.02	0.145^*^	1.01	1.00–1.02	0.065
Time to treatment, h	1.04	0.98–1.11	0.198^*^	1.05	0.95–1.16	0.378
Post-treatment TIMI	1.48	0.90–2.45	0.121^*^	1.22	0.62–2.40	0.568
ADC-thresholded volume	1.24	1.03–1.49	0.025^*^	0.68	0.42–1.10	0.115
ASL reperfusion vol-25	2.07	1.45–2.96	0.001^**^	3.39	1.61–7.17	0.001^**^
IV thrombolysis	1.35	0.40–4.55	0.624	–		
IA thrombolysis	0.53	0.11–2.60	0.431	–		
Clot retrieval	1.88	0.78–4.57	0.161^*^	0.93	0.30–2.83	0.896
Qualitative hyperperfusion	1.01	0.43–2.40	0.976	–		
Relative reperfusion volume	1.39	0.35–5.56	0.646	–		
**PH-2 vs. NO PH-2**						
Atrial fibrillation	1.08	0.30–3.81	0.908	–		
Hypertension	0.77	0.23–2.57	0.675	–		
Diabetes	0.77	0.16–3.80	0.748	–		
Hyperlipidemia	1.77	0.55–5.75	0.340	–		
Aspirin use	1.64	0.45–5.90	0.451	–		
Age, year	1.02	0.97–1.06	0.492	–		
NIHSS	1.07	0.99–1.17	0.086^*^	1.05	0.95–1.17	0.351
Glucose, mg/dL	1.01	0.99–1.02	0.243	–		
Time to treatment, h	0.92	0.76–1.12	0.396	–		
Post-treatment TIMI	1.48	0.73–3.00	0.272	–		
ADC-thresholded volume	1.35	1.06–1.71	0.014^*^	0.89	0.54–1.47	0.649
ASL reperfusion vol-25	2.18	1.42–3.37	0.001^**^	2.32	1.13–4.76	0.022^†^
IV thrombolysis	0.62	0.15–2.55	0.510	–		
IA thrombolysis	1.62	0.31–8.47	0.570	–		
Clot retrieval	3.67	1.10–12.3	0.034^*^	2.33	0.58–9.32	0.233
Qualitative hyperperfusion	0.92	0.29–2.95	0.884	–		
Relative reperfusion volume	0.71	0.11–4.47	0.714	–		

* *P < 0.2 (inclusion in multiple logistic regression)*;

** *P < 0.003125 (significance after Bonferroni correction)*;

† *P < 0.05*.

## Discussion

Patients with acute ischemic stroke treated with intravenous thrombolysis and/or endovascular therapies are at a higher risk for parenchymal hematoma (PH), which has been associated with poor outcomes in prior thrombolysis trials; whereas hemorrhagic infarction (HI) has not. Our research showed that multimodal MR imaging (diffusion and perfusion) can serve as useful markers of undesirable complications after a recanalization procedure. The risk of PH is highly associated with the volume of reperfusion into severely damaged brain tissue, and larger reperfusion volumes may indicate occurrence of higher grade of hemorrhagic transformation.

Lower apparent diffusion coefficient (ADC) values have been associated with greater degrees of ischemia (25, 26) in previous studies. Other studies showed that the percentage of voxels below an ADC value of 550 × 10^−6^ mm^2^/s seems to discriminate between HT- and non-HT-destined lesions (21). These brain tissues are at higher risk for hemorrhagic transformation due to the breakdown of the blood-brain barrier. That may explain why patients with a large volume of ischemia were excluded from thrombolytic or endovascular treatments. However, the size of severe ischemic stroke volume may evolve or reverse (27) dynamically due to many factors, such as treatment effects, baseline collateral grades, and baseline medical conditions. On the other hand, reperfusion appears to be a prerequisite for the development of PH because even if the vasculature is severely damaged within a region of infarct, a PH will not eventuate without the restoration of blood flow (28). Thus, it is necessary to assess and monitor lesion size and reperfusion status post-treatment administration and understand their relations with HT. In this study, 29 patients had post-treatment parenchymal hematoma (PH-1 and PH-2), and 27/29 (93%) patients had successful recanalization (TIMI scores ≥ 2). Thirteen patients had PH-2, and all of these patients had successful recanalization. Although recent randomized clinical trials (MR CLEAN, ESCAPE, EXTEND-IA, SWIFT PRIME, REVASCAT, DIFFUSE III, and DAWN) have consistently shown the efficacy of adding endovascular therapy vs. standard care in treating patients with acute anterior circulation ischemic stroke (3–10), for individual patients, attention should still be paid to prevent high grade of HT, especially after successful recanalization treatments (29).

Reperfusion injury has been demonstrated at molecular and cellular levels in animal models (30). However, its impact in routinely treated clinical stroke cases remains unquantified. In this study, we used different thresholds to define reperfusion within severely damaged brain tissue and found that hyperperfusion is not a prerequisite to cause high grade of HT which was consistent with our previous study that PH did not differ in frequency among patients with and without hyperperfusion (*P* > 0.05) (23). Reperfusion into severely damaged brain tissue at least as high as 25% of its contralateral side best discriminates PH vs. non-PH and PH-2 vs. non-PH-2. This is reasonable because full restoration of blood flow may not be achieved in these tissues due to cell damage and edema, distal microemboli, or extensive damage to the microvasculature despite complete recanalization (31).

Our study also showed that the volume of reperfusion outperformed the volume of severely damaged brain tissue in terms of predicting occurrence of high grade of HT, and the latter can only be used roughly to estimate HT occurrence clinically. This further confirmed the fact that HT is more likely to occur within and/or near severely damaged brain tissue with reperfusion than those without reperfusion. It is worth noting that the hemorrhage may occur adjacent to the ADC thresholded lesion, which might be due to dysfunctional autoregulation as the blood flow approaches the lesion. Increasing volume of reperfusion contributed to the occurrence of higher grade of HT. Patients with ASL reperfusion vol-25 larger than 12 ml were more likely to experience PH-2, and therefore need to be monitored carefully. Blood pressure should be carefully controlled (32, 33), and delayed administration of antithrombotics might be considered, balancing risk vs. benefit (34, 35). It should be noted that the cut-off volume (12 ml) should be interpreted with consideration because this value was determined with methods related to present DWI lesions and may vary in different clinical centers. It is interesting that blood glucose was not an independent predictor of PH as described in previous literature (36). Perhaps because most of the patients in this cohort (71 out of 102 patients) exhibited relatively high blood glucose > 110 mg/dL, blood glucose did not differentiate the outcomes very well. With results from more patients in future analyses, it may be possible to determine if infarct volume and blood glucose are also truly independent predictors of HT.

Finally, although patients with complete recanalization had higher average CBF within the DWI lesion, inhomogeneous reperfusion (volume distribution of different reperfusion status within the DWI-delineated infarct core ranges greatly) existed regardless of recanalization results, which might be due to spontaneous or futile recanalization. This necessitates personalized evaluation of reperfusion and diffusion status after targeted intervention to help discriminate patients at risk for HT from those who may definitely benefit from treatment.

This study has several limitations. First, ASL and DWI imaging were acquired within 24 h post-treatment, and we did not perform multi-MRI examinations to trace the dynamic evolution of the DWI lesion and reperfusion status, which was challenging in clinical settings. Also, two patients had early occurrence of PH-1 and were excluded from the study which could potentially induce a bias. Second, many variables may also contribute to acute hemorrhagic transformation such as poor collateral status, damaged BBB, rCBV, or antithrombotic treatments. Though not currently available in this dataset, we need to include more risk factors to better understand the mechanism of hemorrhagic transformation in future analyses (37–39). Finally, although previous literature showed that TIMI had been predictive of clinical outcome across studies, most recent research used modified TICI (mTICI) to evaluate recanalization because mTICI is superior to TIMI for predicting clinical outcomes after intra-arterial therapy (40). We used TIMI to evaluate recanalization results instead of mTICI because not all the patients had a DSA examination to enable accurate mTICI evaluation.

## Conclusion

Reperfusion into severely damaged brain tissue in AIS patients can be evaluated with multimodal MR examinations. Increasing volume of reperfusion within the DWI-delineated infarct core is highly associated with the occurrence of higher grade of HT.

## Data Availability Statement

The datasets generated for this study are available on request to the corresponding author.

## Ethics Statement

The studies involving human participants were reviewed and approved by UCLA IRB. Written informed consent for participation was not required for this study in accordance with the national legislation and the institutional requirements.

## Author Contributions

SY, SM, and DW contributed conception and design of the study. SY, SM, DL, XQ, and NS organized the database. SM, LY, and DW performed the statistical analysis. SY wrote the first draft of the manuscript. SM, DL, XQ, LY, JS, and NS wrote sections of the manuscript. All authors contributed to manuscript revision, read, and approved the submitted version.

## Conflict of Interest

The authors declare that the research was conducted in the absence of any commercial or financial relationships that could be construed as a potential conflict of interest. The reviewer J-MO declared a past co-authorship with several of the authors DL, JS to the handling editor.
