# Supplementary material for: Reperfusion Into Severely Damaged Brain Tissue Is Associated With Occurrence of Parenchymal Hemorrhage for Acute Ischemic Stroke
Source: Front Neurol. 2020 Jun 26;11:586. doi: 10.3389/fneur.2020.00586 (PMC7332705; doi:10.3389/fneur.2020.00586)
Supplement: Supplementary file 1 [file Table_1.doc]

Supplementary Material

**Supplemental Table I** AUC values for prediction of PH (parenchymal hematoma) and PH-2

| AUC values | ADC threshold lesion volumes | ASL-reperfusion Volumes Percentile Threshold | | |
| --- | --- | --- | --- | --- |
|  |  | 25% | 50% | 75% |
| For prediction of PH | 0.685 | 0.783 | 0.777 | 0.737 |
| For prediction of PH2 | 0.754 | 0.844 | 0.838 | 0.792 |
